# Crystal structure and Hirshfeld surface analysis of the product of the ring-opening reaction of a di­hydro­benzoxazine: 6,6′-[(cyclo­hexyl­aza­nedi­yl)bis­(methyl­ene)]bis­(2,4-di­methyl­phenol)

**DOI:** 10.1107/S2056989020009184

**Published:** 2020-07-10

**Authors:** Suttipong Wannapaiboon, Yuranan Hanlumyuang, Kantapat Chansaenpak, Piyanut Pinyou, Chatchai Veranitisagul, Apirat Laobuthee, Worawat Wattanathana

**Affiliations:** a Synchrotron Light Research Institute, 111 University Avenue, Suranaree, Muang, Nakhon Ratchasima 30000, Thailand; b Department of Materials Engineering, Faculty of Engineering, Kasetsart University 10900, Thailand; c National Nanotechnology Center, National Science and Technology Development Agency, Thailand Science Park, Pathum Thani, 12120, Thailand; d School of Chemistry, Institute of Science, Suranaree University of Technology, 111 University Avenue, Suranaree, Muang, Nakhon Ratchasima 30000, Thailand; e Department of Materials and Metallurgical Engineering, Faculty of Engineering, Rajamangala University of Technology Thanyaburi, Pathumthani 12110, Thailand

**Keywords:** crystal structure, di­hydro­benzoxazine, Hirshfeld surface

## Abstract

In the title unsymmetrical tertiary amine, C_24_H_33_NO_2_, which arose from the ring-opening reaction of a di­hydro­benzoxazine, two 2,4-di­methyl­phenol moieties are linked by a 6,6′-(cyclo­hexyl­aza­nedi­yl)-bis­(methyl­ene) bridge: the dihedral angle between the dimethyl­phenol rings is 72.45 (7)°. The cyclo­hexyl ring adopts a chair conformation with the exocyclic C—N bond in an equatorial orientation. One of the phenol OH groups forms an intra­molecular O—H⋯N hydrogen bond, generating an *S*(6) ring, and a short intra­molecular C—H⋯O contact is also present. In the crystal, O—H⋯O hydrogen bonds link the mol­ecules into *C*(10) chains propagating along the [100] direction. The Hirshfeld surface analysis of the title compound confirms the presence of these intra- and inter­molecular inter­actions. The corresponding fingerprint plots indicate that the most significant contacts in the crystal packing are H⋯H (76.4%), H⋯C/C⋯H (16.3%), and H⋯O/O⋯H (7.2%).

## Abstract 2

In the title unsymmetrical tertiary amine, which arose from the ring-opening reaction of a di­hydro­benzoxazine, two 2,4-di­methyl­phenol moieties are linked by a 6,6′-(cyclo­hexyl­aza­nedi­yl)-bis­(methyl­ene) bridge: the dihedral angle between the phenol rings is 72.45 (7)°. The cyclo­hexyl ring adopts a chair conformation with the exocyclic C—N bond in an equatorial orientation.

## Chemical context   

Di­hydro-benzoxazines contain a benzene ring fused with a di­hydro-oxazine ring (a six-membered heterocycle containing one nitro­gen atom and one oxygen atom). Several isomers of di­hydro-benzoxazines can be formed by varying the heteroatomic positions within the di­hydro-oxazine ring. Among the different isomers of di­hydro-benzoxazines, only 3,4-di­hydro-2*H*-benzo[*e*]-1,3-oxaxines (commonly called 1,3-2*H*-benzoxazine monomers) can undergo a ring-opening polymerization reaction to form polybenzoxazines. As a result of various promising physical and chemical properties, polybenzoxazines have been studied by a number of workers (Ishida & Allen, 1996 ▸; Ishida & Agag 2011 ▸; Kiskan *et al.*, 2011 ▸; Demir *et al.*, 2013 ▸; Kim & & Ishida, 2001 ▸; Velez-Herrera *et al.*, 2008 ▸; Xu *et al.*, 2018 ▸). Moreover, a ring-opening polymerization to form the aza-methyl­ene-phenol [–N*R*–CH_2_–C_6_H_4_(OH)–] moiety provides such hydrogen bonding as to inter­connect with other materials (Froimowicz *et al.*, 2016 ▸; Iguchi *et al.*, 2018 ▸).

Inter­estingly, the use of phenol derivatives as initiators for the ring-opening polymerization of 3,4-di­hydro-2*H*-benzo[*e*]-1,3-oxaxines leads to the formation of small mol­ecules instead of polybenzoxazines (Chirachanchai *et al.*, 2009 ▸). These small mol­ecules (so-called di­hydro-benzoxazine dimers), which generally possess an aza-methyl­ene-phenol group, have been employed as models for describing polybenzoxazines (Hemvichian *et al.*, 2002 ▸). In addition, the asymmetric Mannich reaction of the derivatives of di­hydro-benzoxazine dimers, where only one OH group undergoes the ring-closure reaction has been reported (Laobuthee *et al.*, 2001 ▸). As a result of these aza-methyl­ene-phenol moieties, inter­molecular and intra­molecular hydrogen bonds are found in both the polybenzoxazines and the di­hydro-benzoxazine dimers. They enhance the reactivity of the di­hydro-benzoxazine dimers towards transition and rare-earth metal ions with respect to the common phenolic compounds. For instances, di­hydro-benzoxazine dimers have been reported to be good chelating agents (Iguchi *et al.*, 2018 ▸) for cerium ions (Veranitisagul *et al.*, 2011 ▸) and copper ions (Phongtamrug *et al.*, 2006 ▸).
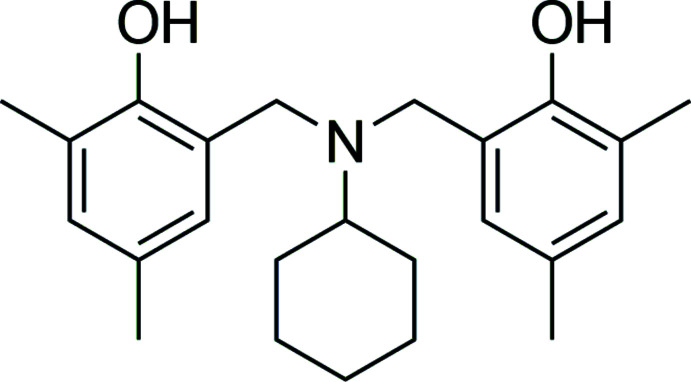


In this work, as part of our ongoing studies in this area (Wattanathana *et al.*, 2016 ▸), we report the synthesis, crystal structure and Hirshfeld surface analysis of the title compound, (I).

## Structural commentary   

**Figure 1:**
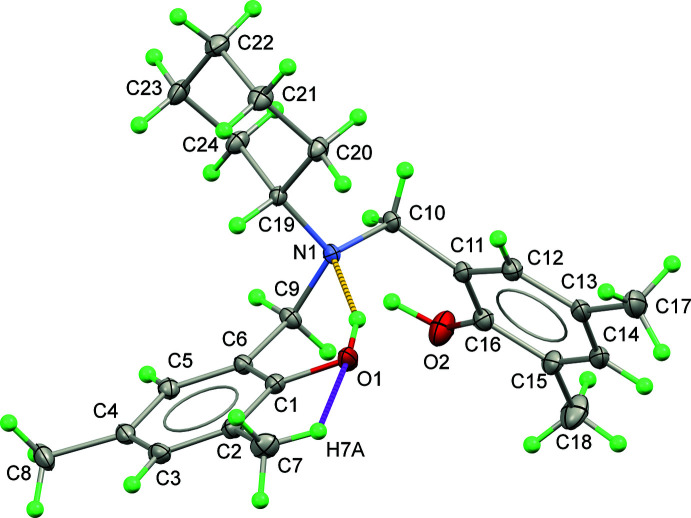
The mol­ecular structure of (I) with displacement ellipsoids drawn at the 50% probability level. The O—H⋯O and C—H⋯O hydrogen bonds are shown as yellow and magenta dashed lines, respectively.

Fig. 1 ▸ shows the mol­ecular structure of (I), which crystallizes in space group *Pna*2_1_. The tertiary-amine nitro­gen atom (N1) adopts a distorted trigonal pyramidal shape because of the expansion of the angles around N1 atom [C9—N1—C19 = 112.59 (15); C10—N1—C9 = 109.97 (15); C10—N1—C19 = 115.09 (15); bond-angle sum = 337.7°].

The non-hydrogen atoms of the 2,4-di­methyl­phenol moieties, namely C1–C8/O1 and C11–C18/O2, are almost planar (r.m.s. deviations = 0.030 and 0.017 Å, respectively) and their mean planes subtend a dihedral angle of 72.45 (7)°. The C atoms in the methyl groups in the *para*-positions with respect to the OH groups deviate the most from the calculated mean planes with deviations of 0.043 (2) for C8 and −0.033 (2) Å for C17. The cyclo­hexyl group adopts a regular chair conformation as seen from the C—C—C bond angles, which are in the range 109.14 (17)° to 111.59 (17)°. The hydrogen atom bonded to C19 (H19) is in the axial position to allow the bulkier group (N1 tertiary-amine nitro­gen atom) to be located at the equatorial position.

According to freely refined positions of the O-bound hydrogen atoms (H1 and H2), H1 points toward N1 to set up an intra­molecular O—H⋯N hydrogen bond with an *S*(6) graph-set motif (Table 1 ▸). This type of intra­molecular O—H⋯N hydrogen bond is commonly noticed in the compounds having –OH and aza­methyl­ene groups attached to the benzene ring in the *ortho* positions (Suramitr *et al.*, 2020 ▸), especially dihydro-benzoxazine dimer derivatives (Veranitisagul *et al.*, 2012 ▸; Wattanathana *et al.*, 2012 ▸, 2016 ▸). In addition to the classical hydrogen bond, one of the hydrogen atoms on the methyl side chain at the ortho position to the O1 atom exhibits a C7—H7*A*⋯O1 close contact (Table 1 ▸) The characteristics of specific interactions for compound (I) are displayed as a non-covalent interaction plot (NCIPLOT) (Johnson *et al.*, 2010 ▸; Contreras-García *et al*., 2011 ▸) in Fig. S1 of the supporting information.

## Supra­molecular features   

**Figure 2:**
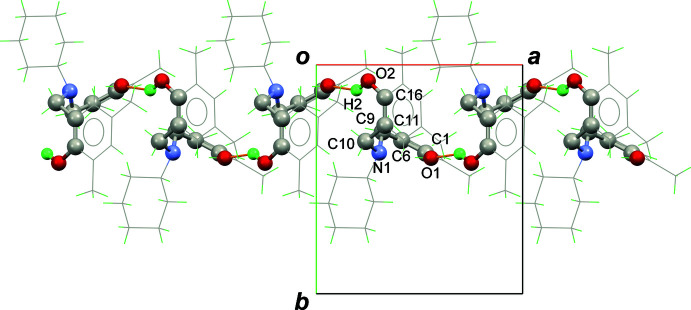
A view down [001] illustrating part of a [100] *C*(10) chain of O—H⋯O hydrogen bonds in the extended structure of (I).

The other (O2) phenol group in (I) forms an inter­molecular O—H⋯O hydrogen bond with O1 as the acceptor, which generates *C*(10) chains in the crystal, propagating in the [100] direction (Fig. 2 ▸). Unlike other dihydro-benzoxazine dimer derivatives, the title compound does not exhibit *R_2_^2^*(20) hydrogen-bonded loops like those formed in 6,6′-(methyl­aza­nedi­yl)bis­(methyl­ene)bis­(2,4-di­methyl­phenol) (NUPJOX: Dunkers *et al.*, 1996 ▸; Phongtamrug *et al.*, 2006 ▸; Veranitisagul *et al.*, 2012*a* ▸), 2,2′-(cyclo­hexyl­aza­nedi­yl)bis­(methyl­ene)bis­(4-ethyl­phenol) (SACYAZ and SADPEV; Wattanathana *et al.*, 2016 ▸), 2,2′-(methyl­aza­nedi­yl)bis­(methyl­ene)bis­(4-methyl­phen­ol) (IDUHEV; Wu *et al.*, 2006 ▸), 2,2′-(methyl­aza­nedi­yl)bis­(meth­yl­ene)bis­(4-meth­oxy­phenol) (XEBBIR; Veranitisagul *et al.*, 2012*b* ▸), 2,2′-(cyclo­hexyl­aza­nedi­yl)bis­(methyl­ene)bis­(4-meth­yl­phenol) (HETGOD; Phongtamrug *et al.*, 2006 ▸), and 2,2′-(cyclo­hexyl­aza­nedi­yl)bis­(methyl­ene)bis­(4-ethyl­phenol) (CEGYUK; Wattanathana *et al.*, 2012 ▸). This might be due to a greater steric effect from both the methyl and cyclo­hexyl groups.

**Figure 3:**
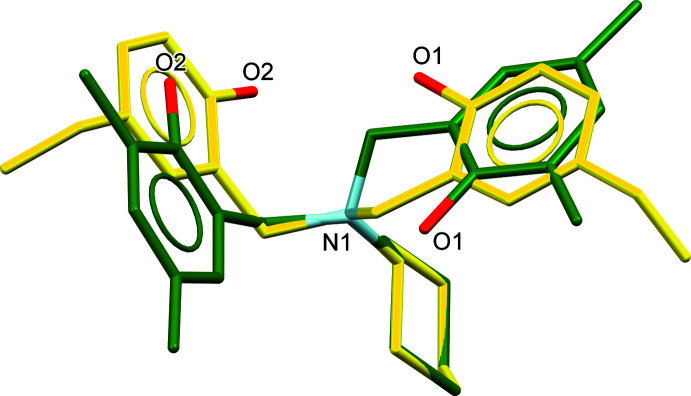
Overlay diagram of (I) (green structure) and its structural isomer (yellow structure, CEGYUK; Wattanathana *et al.*, 2012 ▸). The N and six cyclo­hexyl C atoms are used as centers for structural overlay.

The structure overlay of the title compound (green compound) and its structural isomer with only ethyl groups at the *para*-positions of the phenol rings (CEGYUK; Wattanathana *et al.*, 2012 ▸) is displayed in Fig. 3 ▸. For CEGYUK, both O1 and O2 point toward the same side of the mol­ecule to form the *R_2_^2^*(20) hydrogen-bond motif just mentioned, while the O1 and O2 atoms of (I) are oriented in the opposite direction in order to reduce the steric effect. Therefore, the title mol­ecules are joined together in an end-to-end packing mode into [100] chains (Fig. 2 ▸), where it may be seen that the bulky substituent groups are arrayed in an alternating fashion along the chain.

## Hirshfeld analysis   

**Figure 4:**
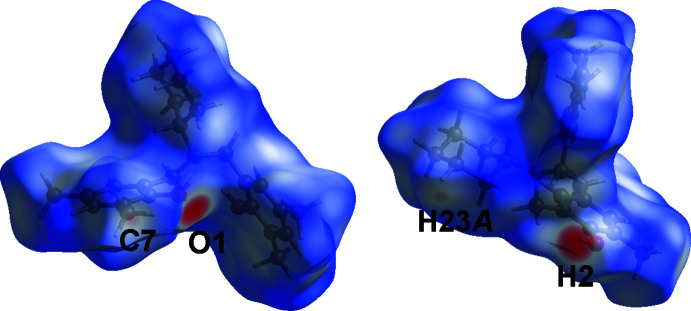
A view of the three-dimensional Hirshfeld surface of (I) plotted over *d*_norm_ in the range −0.56 to 1.39 a.u.

To better understand and visualize the inter­actions within the crystal of the title compound, a Hirshfeld surface (HS) analysis (Spackman & Jayatilaka, 2009 ▸) was carried out using *Crystal Explorer 17.5* software (Turner *et al.*, 2017 ▸). The HS plotted over the given range of *d*_norm_ from −0.56 to 1.39 a.u. (Fig. 4 ▸) shows faint red spots near O1, H2, and C7, confirming the *S*(6) ring, *C*(10) chain, and C—H⋯O inter­action, respectively.

**Figure 5:**
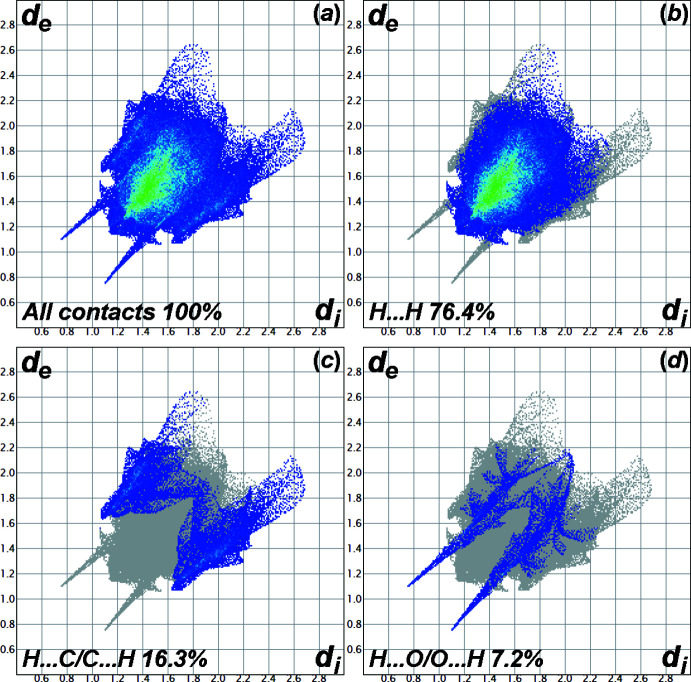
The full two-dimensional fingerprint plots for (I), showing (*a*) all inter­actions, and those delineated into (*b*) H⋯H, (*c*) C⋯H /H⋯C and (*d*) O⋯H/ H⋯O inter­actions.

Fig. 5 ▸ shows the full two-dimensional fingerprint plot and those delineated into individual inter­actions (McKinnon *et al.*, 2007 ▸). The fingerprint plots show that the major contacts in the crystal structure are the contacts regarding H atoms only as the sum of all the H-related contributions is 99.9%. The H⋯H contacts are characterized as a single spike at *d*_e_ + *d*_i_ ≃ 2.3 Å with the contribution of 76.4%, while the H⋯C/C⋯H contacts are observed as a pair of beak-shaped tips at *d*_e_ + *d*_i_ ≃ 2.75 Å with a contribution of 16.3%. The pair of sharp peaks at *d*_e_ + *d*_i_ ≃ 2.2 Å represents the H⋯O/O⋯H contacts (7.2%). The C⋯C contact only participates slightly in the crystal packing as its individual contribution is only 0.1%. The other contacts, *i.e*., N⋯N, H⋯N/N⋯H, C⋯N/N⋯C, C⋯O/O⋯C, show no effect on the crystal packing due to the contribution of 0.0%.

## Database survey   

A search for structures containing the bis­(phenol) linked by a bis­(methyl­ene)aza bridge in the Cambridge Structural Database (CSD version 5.41, November 2019 + two updates; Groom *et al.*, 2016 ▸) showed 156 match entries. Structural diversity of the dihydro-benzoxazine derivatives is observed as a result of the variation of the substituent groups on both the phenol moieties and tertiary-amine nitro­gen atom. Several crystal structures of dihydro-benzoxazine dimer derivatives with no other substituent groups on both the phenol rings have been reported (BUZWUP; Abrahams *et al.*, 2009 ▸; KEJRAU; Kuźnik *et al.*, 2012 ▸). The crystal structures of dihydro-benzoxazine dimer derivatives with *ortho* substituents have also been reported, *e.g*., *tert*-butyl substituents (CIJLEN; Kelly *et al.*, 2007 ▸) and meth­oxy substituents (SILROV; Liu *et al.*, 2007 ▸). However, no crystal structures of dihydro-benzoxazine dimers possessing *meta* substituents have been reported. This might be due to the *ortho* and *para* directing property of the phenolic –OH groups. Dihydro-benzoxazine dimer derivatives with *para* substituents are very common, *viz*. with methyl groups (FANHOT; Janas *et al.*, 2012 ▸, Singh *et al.*, 2012 ▸; HETGOD; Phongtamrug *et al.*, 2006 ▸; ICEMIO; Wang *et al.*, 2011*a* ▸, Rivera & Bolte, 2016 ▸; IDUHEV; Wu *et al.*, 2006 ▸; USODAC; Wang *et al.*, 2011*b* ▸,*c* ▸), ethyl groups (CEGYUK; Wattanathana *et al.*, 2012 ▸, SACYAZ and SADPEV; Wattanathana *et al.*, 2016 ▸), a meth­oxy group (XEBBIR; Veranitisagul *et al.*, 2012*b* ▸), and *tert*-butyl groups (GIKJOC; Redjel *et al.*, 2018 ▸). Apart from the monosubstituted derivatives, there are some reports on the crystal structures of *ortho* and *para* disubstituted derivatives, *e.g*., HEPZOU (Zhang *et al.*, 2018 ▸) and RACMEP (Lionetti *et al.*, 2010 ▸). Moreover, dihydro-benzoxazine dimers can also have different substituents on both the phenol rings as in AMEFUT, AMEGAA and AMEGEE (Sony *et al.*, 2003 ▸), resulting in considerable structural variety.

When more restriction is applied to the search of 2,4-di­methyl­bis­(phenol) linked by bis­(methyl­ene)aza bridge, the number of match structures is now reduced to 38 hits as only the N-substituted grouping can change. Examples of different N-substituents of the 6,6′-(aza­nedi­yl)bis­(methyl­ene)bis­(2,4-di­methyl­phenol) derivatives are –CH_3_ (NUPJOX; Dunkers *et al.*, 1996 ▸, Phongtamrug *et al.*, 2006 ▸, Veranitisagul *et al.*, 2012*a* ▸), –CH_2_CH_2_OCH_3_ (CAKDUP; Hasan *et al.*, 2011 ▸), –CH_2_CH_2_N(CH_3_)_2_ (ESAHUB; Velusamy *et al.*, 2003 ▸, Lorber *et al.*, 2005 ▸), –CH_2_CH_2_CH_2_OH (GIMWIL; Olesiejuk *et al.*, 2018 ▸), –CH_2_CH_2_CH_2_Cl (GIMWOR; Olesiejuk *et al.*, 2018 ▸), –CH_2_CH_2_N(CH_2_CH_3_)_2_ (TOJSUI; Singh *et al.*, 2012 ▸), and –CH_2_CH_2_CH_2_N(CH_3_)_2_ (ZUXJAF; Bowser *et al.*, 2016 ▸).

## Synthesis, characterization and crystallization   

Firstly, the corresponding dihydro-benzoxazine monomer, 3-cyclo­hexyl-6,8-dimethyl-3,4-di­hydro-*2H*-benzo[*e*][1,3]oxa­zine, was prepared by a one-pot Mannich reaction (Chirachanchai *et al.*, 2009 ▸; Wattanathana *et al.*, 2014 ▸). Cyclo­hexyl­amine (0.99 g, 10 mmol), paraformaldehyde (0.63 g, 20 mmol) and 2,4-di­methyl­phenol (1.22 g, 10 mmol) were dissolved in dioxane (10 ml). The mixture was refluxed for 6 h to obtain a clear yellow solution. The solvent was removed by a rotary evaporator to obtain a yellowish viscous liquid as a crude product. After that, 10 ml of di­chloro­methane were added to the dried crude product. The crude product was then washed by a liquid–liquid extraction method using 3 *N* NaOH (10 ml) three times, followed by 10 ml of deionized water for three more times until the solution became neutral. The product was dried by anhydrous sodium sulfate. Then, the di­chloro­methane solvent was removed by a rotary evaporator and consequently the dihydro-benzoxazine monomer, 3-cyclo­hexyl-6,8-dimethyl-3,4-di­hydro-*2H*-benzo[*e*][1,3]oxazine, (II), was collected.

An equimolar amount of 2,4-di­methyl­phenol was then mixed with (II) and the mixture was heated at 333 K overnight. After the reaction was complete, the yellow viscous liquid turned into a yellow solid, which was washed using diethyl ether, giving rise to a white precipitate of the title compound, which was separated from the yellow solution by deca­ntation and rinsing with diethyl ether. The white precip­itate was recrystallized from propan-2-ol solution to yield colourless blocks of (I).

M.p. 425 K; FTIR (KBR pellet, cm^−1^): 3384 (*br*, O—H), 1484 (*vs*, C_a_—C_a_), 1451 (*m*, N–CH_3_), 1245 (*m*, C—N), 1199 (*m*, C—N—C), 858 (*m*, C—N—C); Raman (cm^−1^): 3023 (*m*, C_a_—H), 2942 (*vs*, C*sp* ^3^—H), 1447 (*m*, N—CH_3_); ^1^H NMR (*δ*_H_, ppm): 1.06–1.14 (*m*, 1H), 1.19 (*q*, *J* = 12.0 Hz, 2H), 1.44 (*q*, *J* = 9.5 Hz, 2H), 1.64 (*d*, *J* = 12.0 Hz, 1H), 1.81 (*d*, *J* = 13.0 Hz, 2H), 1.94 (*d*, *J* = 11.5 Hz, 2H), 2.21 (*d*, *J* = 11.0 Hz, 12H), 2.72 (*tt*, *J* = 12.0, 3.0 Hz, 1H), 3.73 (*s*, 4H), 6.70 (*s*, 2H), 6.85 (*s*, 2H), 8.04 (*s*, 2H); ^13^C NMR (*δ*_C_, ppm): 16.03 (–CH_3_), 20.61 (–CH_3_), 25.99 (C_cy_), 26.35 (C_cy_), 27.66 (C_cy_), 51.64 (–CH_2_–N*R*_2_), 57.65 (C_cy_—N*R*_2_), 122.01 (C_a_), 124.94 (C_a_), 128.57 (C_a_), 128.65 (C_a_), 131.03 (C_a_), 152.27 (C—OH) (cy = cyclo­hexyl, a = aromatic). Elemental analysis: analysis calculated for C_24_H_33_NO_2_ (%): C 78.47; H 8.99; N 3.82; found: C 78.49; H 8.97; N 3.85. The good agreement (see Fig. S2 in the supporting information) between the measured PXRD pattern of (I) and the calculated pattern based on the single crystal data indicates the high degree of crystal homogeneity and crystallinity of the obtained compound. For full details of the spectroscopic and powder diffraction measurements, see the supporting information.

## Refinement   

Crystal data, data collection and structure refinement details are summarized in Table 2 ▸. The O-bound H atoms (H1 and H2) were located in a difference map and their positions were freely refined. The C-bound H atoms were placed in idealized positions (C—H = 0.95–1.00 Å depending on hybridization) and refined as riding atoms. The methyl groups were allowed to rotate, but not to tip, to best fit the electron density. The constraint *U*_iso_(H) = 1.2*U*_eq_(carrier) or 1.5*U*_eq_(methyl C) was applied in all cases. The absolute structure of (I) was indeterminate in the present refinement.

## Supplementary Material

Crystal structure: contains datablock(s) I, global. DOI: 10.1107/S2056989020009184/hb7926sup1.cif

Structure factors: contains datablock(s) I. DOI: 10.1107/S2056989020009184/hb7926Isup2.hkl

Non-covalent interaction plot and powder diffraction plot. DOI: 10.1107/S2056989020009184/hb7926sup3.docx

Supporting information file. DOI: 10.1107/S2056989020009184/hb7926Isup4.cml

CCDC reference: 2014264

Additional supporting information:  crystallographic information; 3D view; checkCIF report

## Figures and Tables

**Table 1:** Hydrogen-bond geometry (Å, °)

*D*—H⋯*A*	*D*—H	H⋯*A*	*D*⋯*A*	*D*—H⋯*A*
O1—H1⋯N1	0.89 (4)	1.81 (4)	2.630 (2)	153 (3)
O2—H2⋯O1^i^	0.99 (4)	1.87 (4)	2.741 (2)	145 (3)
C7—H7*A*⋯O1	0.98	2.40	2.854 (3)	108

Symmetry code: (i) 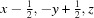.

**Table 2:** Experimental details

Crystal data	
Chemical formula	C_24_H_33_NO_2_
*M*_r_	367.51
Crystal system, space group	Orthorhombic, *P* *n* *a*2_1_
Temperature (K)	100
*a*, *b*, *c* (Å)	10.2778 (7), 11.4064 (11), 17.5586 (15)
*V* (Å^3^)	2058.4 (3)
*Z*	4
Radiation type	Mo *K*α
μ (mm^−1^)	0.07
Crystal size (mm)	0.50 × 0.28 × 0.22

Data collection	
Diffractometer	Bruker APEXII CCD
Absorption correction	Multi-scan (*SADABS*; Bruker, 2016 ▸)
*T*_min_, *T*_max_	0.661, 0.747
No. of measured, independent and observed [*I* > 2σ(*I*)] reflections	17289, 7626, 6367
*R*_int_	0.035
(sin θ/λ)_max_ (Å^−1^)	0.771

Refinement	
*R*[*F* ^2^ > 2σ(*F* ^2^)], *wR*(*F* ^2^), *S*	0.053, 0.139, 1.04
No. of reflections	7626
No. of parameters	256
No. of restraints	1
H-atom treatment	H atoms treated by a mixture of independent and constrained refinement
Δρ_max_, Δρ_min_ (e Å^−3^)	0.85, −0.26

Computer programs: *APEX2* and *SAINT* (Bruker, 2018 ▸), *SHELXT* (Sheldrick, 2015*a* ▸), *SHELXL* (Sheldrick, 2015*b* ▸), *OLEX2* (Dolomanov *et al.*, 2009 ▸), *Mercury* (Macrae *et al.*, 2020 ▸) and *publCIF* (Westrip, 2010 ▸).

## supplementary crystallographic information

### Crystal data

**Table:**

C_24_H_33_NO_2_	*D*_x_ = 1.186 Mg m^−^^3^
*M**_r_* = 367.51	Mo *K*α radiation, λ = 0.71073 Å
Orthorhombic, *P**n**a*2_1_	Cell parameters from 4297 reflections
*a* = 10.2778 (7) Å	θ = 2.3–30.4°
*b* = 11.4064 (11) Å	µ = 0.07 mm^−^^1^
*c* = 17.5586 (15) Å	*T* = 100 K
*V* = 2058.4 (3) Å^3^	Block, colourless
*Z* = 4	0.50 × 0.28 × 0.22 mm
*F*(000) = 800	

### Data collection

**Table:**

Bruker APEXII CCD diffractometer	6367 reflections with *I* > 2σ(*I*)
Graphite monochromator	*R*_int_ = 0.035
φ and ω scans	θ_max_ = 33.2°, θ_min_ = 2.7°
Absorption correction: multi-scan (SADABS; Bruker, 2016)	*h* = −15→13
*T*_min_ = 0.661, *T*_max_ = 0.747	*k* = −17→16
17289 measured reflections	*l* = −26→26
7626 independent reflections	

### Refinement

**Table:**

Refinement on *F*^2^	Primary atom site location: dual
Least-squares matrix: full	Hydrogen site location: mixed
*R*[*F*^2^ > 2σ(*F*^2^)] = 0.053	H atoms treated by a mixture of independent and constrained refinement
*wR*(*F*^2^) = 0.139	*w* = 1/[σ^2^(*F*_o_^2^) + (0.0816*P*)^2^ + 0.0042*P*] where *P* = (*F*_o_^2^ + 2*F*_c_^2^)/3
*S* = 1.04	(Δ/σ)_max_ < 0.001
7626 reflections	Δρ_max_ = 0.85 e Å^−^^3^
256 parameters	Δρ_min_ = −0.26 e Å^−^^3^
1 restraint	

### Special details

**Table:**

Geometry. All esds (except the esd in the dihedral angle between two l.s. planes) are estimated using the full covariance matrix. The cell esds are taken into account individually in the estimation of esds in distances, angles and torsion angles; correlations between esds in cell parameters are only used when they are defined by crystal symmetry. An approximate (isotropic) treatment of cell esds is used for estimating esds involving l.s. planes.

### Fractional atomic coordinates and isotropic or equivalent isotropic displacement parameters (Å^2^)

**Table:**

	*x*	*y*	*z*	*U*_iso_*/*U*_eq_	
O1	0.55606 (15)	0.40779 (15)	0.48635 (9)	0.0185 (3)	
O2	0.25137 (17)	0.07066 (16)	0.38097 (11)	0.0282 (4)	
N1	0.30494 (16)	0.38434 (15)	0.46177 (9)	0.0124 (3)	
C11	0.33237 (18)	0.26439 (18)	0.34561 (11)	0.0146 (3)	
C1	0.53009 (19)	0.38603 (17)	0.56149 (11)	0.0136 (3)	
C4	0.4756 (2)	0.34173 (18)	0.71588 (11)	0.0162 (4)	
C16	0.33219 (19)	0.14251 (19)	0.34158 (12)	0.0163 (4)	
C6	0.41375 (18)	0.33056 (18)	0.58231 (10)	0.0129 (3)	
C5	0.3869 (2)	0.31068 (18)	0.65887 (11)	0.0148 (3)	
H5	0.306648	0.275282	0.672746	0.018*	
C9	0.31994 (19)	0.29297 (18)	0.52109 (11)	0.0148 (4)	
H9A	0.351767	0.219736	0.497244	0.018*	
H9B	0.234101	0.276476	0.544316	0.018*	
C19	0.24654 (17)	0.49405 (17)	0.49191 (11)	0.0118 (3)	
H19	0.290794	0.509791	0.541586	0.014*	
C10	0.23905 (18)	0.33456 (18)	0.39420 (11)	0.0144 (3)	
H10A	0.201570	0.398953	0.363412	0.017*	
H10B	0.166781	0.283321	0.410990	0.017*	
C3	0.5912 (2)	0.39448 (18)	0.69330 (12)	0.0170 (4)	
H3	0.653425	0.414046	0.731218	0.020*	
C24	0.10054 (19)	0.48969 (19)	0.50922 (12)	0.0170 (4)	
H24A	0.081486	0.423062	0.543590	0.020*	
H24B	0.051024	0.478236	0.461434	0.020*	
C14	0.5057 (2)	0.1484 (2)	0.24962 (12)	0.0201 (4)	
H14	0.564743	0.108269	0.217144	0.024*	
C13	0.50935 (19)	0.2702 (2)	0.25194 (12)	0.0182 (4)	
C12	0.4226 (2)	0.32580 (19)	0.30100 (12)	0.0167 (4)	
H12	0.424682	0.408903	0.304371	0.020*	
C2	0.61996 (19)	0.42005 (18)	0.61731 (12)	0.0153 (4)	
C20	0.27874 (19)	0.59824 (18)	0.44034 (12)	0.0165 (4)	
H20A	0.373603	0.600307	0.430636	0.020*	
H20B	0.233910	0.588665	0.390821	0.020*	
C15	0.4194 (2)	0.08345 (19)	0.29282 (13)	0.0191 (4)	
C22	0.09195 (19)	0.7111 (2)	0.49721 (13)	0.0199 (4)	
H22A	0.039773	0.707735	0.449863	0.024*	
H22B	0.068791	0.784064	0.524558	0.024*	
C21	0.2364 (2)	0.71323 (18)	0.47706 (13)	0.0187 (4)	
H21A	0.253598	0.778781	0.441510	0.022*	
H21B	0.288055	0.726786	0.523825	0.022*	
C23	0.0594 (2)	0.60523 (19)	0.54719 (13)	0.0197 (4)	
H23A	−0.035431	0.603583	0.557053	0.024*	
H23B	0.104440	0.613096	0.596767	0.024*	
C7	0.7421 (2)	0.4850 (2)	0.59612 (14)	0.0216 (4)	
H7A	0.752890	0.483810	0.540676	0.032*	
H7B	0.817154	0.447173	0.620209	0.032*	
H7C	0.735771	0.566363	0.613687	0.032*	
C8	0.4433 (2)	0.3241 (2)	0.79879 (12)	0.0228 (4)	
H8A	0.412900	0.398284	0.820627	0.034*	
H8B	0.521270	0.297887	0.826066	0.034*	
H8C	0.374907	0.264769	0.803607	0.034*	
C17	0.6012 (2)	0.3382 (3)	0.20141 (13)	0.0265 (5)	
H17A	0.681173	0.292866	0.193814	0.040*	
H17B	0.622402	0.413246	0.225565	0.040*	
H17C	0.559676	0.352574	0.152043	0.040*	
C18	0.4156 (3)	−0.0484 (2)	0.28772 (17)	0.0318 (5)	
H18A	0.418848	−0.081899	0.339082	0.048*	
H18B	0.490459	−0.076175	0.258236	0.048*	
H18C	0.334966	−0.072901	0.262519	0.048*	
H1	0.480 (3)	0.405 (3)	0.463 (2)	0.042 (10)*	
H2	0.195 (4)	0.111 (4)	0.419 (3)	0.057 (12)*	

### Atomic displacement parameters (Å^2^)

**Table:**

	*U*^11^	*U*^22^	*U*^33^	*U*^12^	*U*^13^	*U*^23^
O1	0.0135 (6)	0.0288 (8)	0.0133 (6)	0.0015 (6)	0.0020 (5)	0.0010 (6)
O2	0.0312 (9)	0.0236 (8)	0.0299 (9)	−0.0038 (7)	0.0126 (7)	−0.0037 (7)
N1	0.0136 (7)	0.0141 (7)	0.0094 (6)	0.0016 (6)	−0.0021 (5)	−0.0020 (5)
C11	0.0150 (8)	0.0172 (9)	0.0116 (7)	0.0026 (7)	−0.0019 (6)	−0.0034 (7)
C1	0.0133 (8)	0.0144 (8)	0.0132 (8)	0.0025 (7)	0.0000 (6)	−0.0009 (7)
C4	0.0221 (9)	0.0144 (9)	0.0120 (8)	0.0034 (7)	−0.0018 (7)	−0.0004 (7)
C16	0.0165 (8)	0.0175 (9)	0.0148 (8)	0.0002 (7)	−0.0016 (6)	−0.0024 (7)
C6	0.0135 (8)	0.0136 (8)	0.0116 (8)	0.0013 (6)	−0.0021 (6)	−0.0007 (6)
C5	0.0176 (8)	0.0130 (8)	0.0138 (8)	0.0008 (7)	0.0000 (6)	0.0004 (7)
C9	0.0165 (8)	0.0153 (9)	0.0125 (8)	−0.0014 (7)	−0.0028 (6)	0.0001 (7)
C19	0.0122 (7)	0.0128 (8)	0.0104 (7)	0.0005 (6)	0.0012 (6)	−0.0010 (6)
C10	0.0149 (8)	0.0169 (9)	0.0115 (7)	0.0019 (7)	−0.0029 (6)	−0.0044 (7)
C3	0.0175 (8)	0.0178 (9)	0.0158 (8)	0.0036 (7)	−0.0057 (7)	−0.0038 (7)
C24	0.0143 (8)	0.0166 (9)	0.0200 (9)	0.0006 (7)	0.0045 (7)	−0.0001 (7)
C14	0.0196 (9)	0.0279 (11)	0.0127 (8)	0.0080 (8)	−0.0023 (7)	−0.0046 (8)
C13	0.0174 (8)	0.0263 (10)	0.0110 (8)	0.0036 (8)	−0.0007 (7)	−0.0007 (8)
C12	0.0193 (9)	0.0171 (9)	0.0135 (8)	0.0021 (7)	−0.0019 (7)	−0.0013 (7)
C2	0.0132 (8)	0.0156 (9)	0.0170 (8)	0.0013 (7)	−0.0016 (6)	−0.0026 (7)
C20	0.0170 (8)	0.0158 (9)	0.0166 (8)	0.0002 (7)	0.0048 (7)	0.0016 (7)
C15	0.0230 (9)	0.0175 (9)	0.0167 (9)	0.0047 (8)	−0.0019 (7)	−0.0057 (8)
C22	0.0193 (9)	0.0178 (9)	0.0224 (10)	0.0047 (7)	0.0018 (7)	−0.0010 (7)
C21	0.0203 (9)	0.0146 (9)	0.0210 (9)	−0.0008 (7)	0.0026 (7)	0.0003 (7)
C23	0.0179 (9)	0.0205 (10)	0.0209 (10)	0.0034 (7)	0.0079 (7)	−0.0023 (8)
C7	0.0156 (9)	0.0235 (10)	0.0256 (10)	−0.0021 (8)	−0.0027 (8)	−0.0032 (8)
C8	0.0334 (11)	0.0237 (10)	0.0113 (8)	0.0018 (9)	−0.0031 (8)	0.0007 (8)
C17	0.0234 (10)	0.0382 (13)	0.0178 (10)	0.0042 (10)	0.0039 (8)	0.0044 (9)
C18	0.0414 (14)	0.0193 (11)	0.0348 (13)	0.0026 (10)	0.0087 (11)	−0.0083 (10)

### Geometric parameters (Å, º)

**Table:**

O1—C1	1.369 (2)	C14—H14	0.9500
O1—H1	0.89 (4)	C14—C13	1.391 (3)
O2—C16	1.356 (3)	C14—C15	1.382 (3)
O2—H2	0.99 (4)	C13—C12	1.393 (3)
N1—C9	1.482 (3)	C13—C17	1.510 (3)
N1—C19	1.485 (2)	C12—H12	0.9500
N1—C10	1.479 (2)	C2—C7	1.504 (3)
C11—C16	1.392 (3)	C20—H20A	0.9900
C11—C10	1.513 (3)	C20—H20B	0.9900
C11—C12	1.402 (3)	C20—C21	1.525 (3)
C1—C6	1.401 (3)	C15—C18	1.507 (3)
C1—C2	1.402 (3)	C22—H22A	0.9900
C4—C5	1.399 (3)	C22—H22B	0.9900
C4—C3	1.390 (3)	C22—C21	1.526 (3)
C4—C8	1.506 (3)	C22—C23	1.530 (3)
C16—C15	1.411 (3)	C21—H21A	0.9900
C6—C5	1.391 (3)	C21—H21B	0.9900
C6—C9	1.506 (3)	C23—H23A	0.9900
C5—H5	0.9500	C23—H23B	0.9900
C9—H9A	0.9900	C7—H7A	0.9800
C9—H9B	0.9900	C7—H7B	0.9800
C19—H19	1.0000	C7—H7C	0.9800
C19—C24	1.532 (3)	C8—H8A	0.9800
C19—C20	1.530 (3)	C8—H8B	0.9800
C10—H10A	0.9900	C8—H8C	0.9800
C10—H10B	0.9900	C17—H17A	0.9800
C3—H3	0.9500	C17—H17B	0.9800
C3—C2	1.397 (3)	C17—H17C	0.9800
C24—H24A	0.9900	C18—H18A	0.9800
C24—H24B	0.9900	C18—H18B	0.9800
C24—C23	1.536 (3)	C18—H18C	0.9800
			
C1—O1—H1	106 (2)	C13—C12—C11	122.80 (19)
C16—O2—H2	115 (2)	C13—C12—H12	118.6
C9—N1—C19	112.59 (15)	C1—C2—C7	120.89 (19)
C10—N1—C9	109.97 (15)	C3—C2—C1	118.06 (18)
C10—N1—C19	115.09 (15)	C3—C2—C7	121.03 (18)
C16—C11—C10	123.80 (18)	C19—C20—H20A	109.5
C16—C11—C12	118.14 (18)	C19—C20—H20B	109.5
C12—C11—C10	118.05 (18)	H20A—C20—H20B	108.1
O1—C1—C6	119.99 (17)	C21—C20—C19	110.85 (17)
O1—C1—C2	119.69 (18)	C21—C20—H20A	109.5
C6—C1—C2	120.32 (18)	C21—C20—H20B	109.5
C5—C4—C8	120.95 (19)	C16—C15—C18	119.7 (2)
C3—C4—C5	117.54 (19)	C14—C15—C16	119.01 (19)
C3—C4—C8	121.43 (19)	C14—C15—C18	121.2 (2)
O2—C16—C11	125.33 (19)	H22A—C22—H22B	108.0
O2—C16—C15	114.21 (19)	C21—C22—H22A	109.4
C11—C16—C15	120.46 (19)	C21—C22—H22B	109.4
C1—C6—C9	119.24 (17)	C21—C22—C23	111.00 (18)
C5—C6—C1	119.68 (18)	C23—C22—H22A	109.4
C5—C6—C9	121.08 (18)	C23—C22—H22B	109.4
C4—C5—H5	119.3	C20—C21—C22	111.22 (17)
C6—C5—C4	121.38 (19)	C20—C21—H21A	109.4
C6—C5—H5	119.3	C20—C21—H21B	109.4
N1—C9—C6	111.60 (16)	C22—C21—H21A	109.4
N1—C9—H9A	109.3	C22—C21—H21B	109.4
N1—C9—H9B	109.3	H21A—C21—H21B	108.0
C6—C9—H9A	109.3	C24—C23—H23A	109.3
C6—C9—H9B	109.3	C24—C23—H23B	109.3
H9A—C9—H9B	108.0	C22—C23—C24	111.59 (17)
N1—C19—H19	106.2	C22—C23—H23A	109.3
N1—C19—C24	116.06 (16)	C22—C23—H23B	109.3
N1—C19—C20	110.85 (15)	H23A—C23—H23B	108.0
C24—C19—H19	106.2	C2—C7—H7A	109.5
C20—C19—H19	106.2	C2—C7—H7B	109.5
C20—C19—C24	110.76 (16)	C2—C7—H7C	109.5
N1—C10—C11	111.41 (15)	H7A—C7—H7B	109.5
N1—C10—H10A	109.3	H7A—C7—H7C	109.5
N1—C10—H10B	109.3	H7B—C7—H7C	109.5
C11—C10—H10A	109.3	C4—C8—H8A	109.5
C11—C10—H10B	109.3	C4—C8—H8B	109.5
H10A—C10—H10B	108.0	C4—C8—H8C	109.5
C4—C3—H3	118.5	H8A—C8—H8B	109.5
C4—C3—C2	122.94 (18)	H8A—C8—H8C	109.5
C2—C3—H3	118.5	H8B—C8—H8C	109.5
C19—C24—H24A	109.9	C13—C17—H17A	109.5
C19—C24—H24B	109.9	C13—C17—H17B	109.5
C19—C24—C23	109.14 (17)	C13—C17—H17C	109.5
H24A—C24—H24B	108.3	H17A—C17—H17B	109.5
C23—C24—H24A	109.9	H17A—C17—H17C	109.5
C23—C24—H24B	109.9	H17B—C17—H17C	109.5
C13—C14—H14	118.8	C15—C18—H18A	109.5
C15—C14—H14	118.8	C15—C18—H18B	109.5
C15—C14—C13	122.5 (2)	C15—C18—H18C	109.5
C14—C13—C12	117.1 (2)	H18A—C18—H18B	109.5
C14—C13—C17	120.9 (2)	H18A—C18—H18C	109.5
C12—C13—C17	122.0 (2)	H18B—C18—H18C	109.5
C11—C12—H12	118.6		
			
O1—C1—C6—C5	−178.81 (18)	C19—C24—C23—C22	57.3 (2)
O1—C1—C6—C9	1.4 (3)	C19—C20—C21—C22	−55.9 (2)
O1—C1—C2—C3	−178.87 (18)	C10—N1—C9—C6	166.26 (16)
O1—C1—C2—C7	2.7 (3)	C10—N1—C19—C24	53.1 (2)
O2—C16—C15—C14	179.63 (19)	C10—N1—C19—C20	−74.3 (2)
O2—C16—C15—C18	0.6 (3)	C10—C11—C16—O2	−1.2 (3)
N1—C19—C24—C23	174.32 (17)	C10—C11—C16—C15	177.75 (18)
N1—C19—C20—C21	−171.58 (15)	C10—C11—C12—C13	−177.36 (18)
C11—C16—C15—C14	0.5 (3)	C3—C4—C5—C6	0.7 (3)
C11—C16—C15—C18	−178.5 (2)	C24—C19—C20—C21	58.1 (2)
C1—C6—C5—C4	−2.0 (3)	C14—C13—C12—C11	−1.4 (3)
C1—C6—C9—N1	−43.1 (2)	C13—C14—C15—C16	−0.5 (3)
C4—C3—C2—C1	−2.8 (3)	C13—C14—C15—C18	178.5 (2)
C4—C3—C2—C7	175.6 (2)	C12—C11—C16—O2	179.99 (19)
C16—C11—C10—N1	106.6 (2)	C12—C11—C16—C15	−1.0 (3)
C16—C11—C12—C13	1.5 (3)	C12—C11—C10—N1	−74.6 (2)
C6—C1—C2—C3	1.5 (3)	C2—C1—C6—C5	0.8 (3)
C6—C1—C2—C7	−176.93 (19)	C2—C1—C6—C9	−178.92 (18)
C5—C4—C3—C2	1.8 (3)	C20—C19—C24—C23	−58.2 (2)
C5—C6—C9—N1	137.12 (19)	C15—C14—C13—C12	0.9 (3)
C9—N1—C19—C24	−74.0 (2)	C15—C14—C13—C17	−177.40 (19)
C9—N1—C19—C20	158.55 (16)	C21—C22—C23—C24	−55.9 (2)
C9—N1—C10—C11	−79.0 (2)	C23—C22—C21—C20	54.7 (2)
C9—C6—C5—C4	177.78 (19)	C8—C4—C5—C6	177.6 (2)
C19—N1—C9—C6	−64.0 (2)	C8—C4—C3—C2	−175.1 (2)
C19—N1—C10—C11	152.53 (16)	C17—C13—C12—C11	176.86 (19)

### Hydrogen-bond geometry (Å, º)

**Table:**

*D*—H···*A*	*D*—H	H···*A*	*D*···*A*	*D*—H···*A*
O1—H1···N1	0.89 (4)	1.81 (4)	2.630 (2)	153 (3)
O2—H2···O1^i^	0.99 (4)	1.87 (4)	2.741 (2)	145 (3)
C7—H7*A*···O1	0.98	2.40	2.854 (3)	108

Symmetry code: (i) *x*−1/2, −*y*+1/2, *z*.
