# Supplementary material for: Crystal structure and Hirshfeld surface analysis of the product of the ring-opening reaction of a di­hydro­benzoxazine: 6,6′-[(cyclo­hexyl­aza­nedi­yl)bis­(methyl­ene)]bis­(2,4-di­methyl­phenol)
Source: Acta Crystallogr E Crystallogr Commun. 2020 Jul 10;76(Pt 8):1239–44. doi: 10.1107/S2056989020009184 (PMC7405559; doi:10.1107/S2056989020009184)
Supplement: Supplementary file 3 [file e-76-01239-sup3.docx]

# Supporting information for

# Crystal structure, Hirshfeld surface analysis and non-covalent interactions of a benzoxazine dimer derivative: 6,6'-(cyclohexylazanediyl)bis(methylene)bis(2,4-dimethylphenol)

# Suttipong Wannapaiboon^1^, Yuranan Hanlumyuang^2^, Kantapat Chansaenpak^3^, Piyanut Pinyou^4^,^­^ Chatchai Veranitisagul^5^, Apirat Laobuthee^2^, and Worawat Wattanathana^2,*^

*^1^Synchrotron Light Research Institute, 111 University Avenue, Suranaree, Muang, Nakhon Ratchasima 30000, Thailand*

*^2^Department of Materials Engineering, Faculty of Engineering, Kasetsart University 10900, Thailand*

*^3^National Nanotechnology Center, National Science and Technology Development Agency, Thailand Science Park, Pathum Thani, 12120, Thailand*

*^4^School of Chemistry, Institute of Science, Suranaree University of Technology, 111 University Avenue, Suranaree, Muang, Nakhon Ratchasima 30000, Thailand*

*^5^Department of Materials and Metallurgical Engineering, Faculty of Engineering, Rajamangala University of Technology Thanyaburi, Pathumthani 12110, Thailand*

*Corresponding author. *E-mail address*: fengwwwa@ku.ac.th (W. Wattanathana)


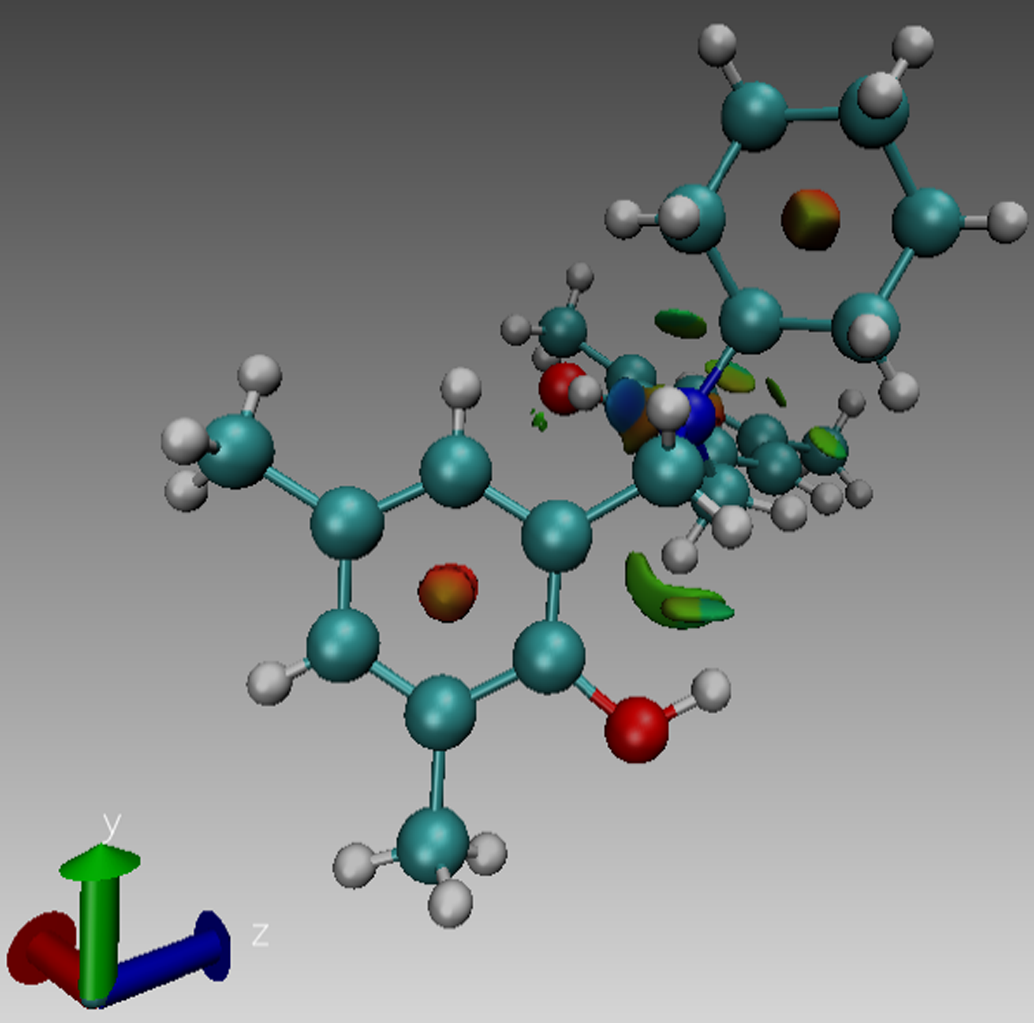


**Fig. S1** Non-covalent inter­action plot (*NCIPLOT*) of the title mol­ecule.

***NCIPLOT* view of the title compound (I) visualized by Visual Molecular Dynamics (VMD) software (Humphrey *et al.*, 1996):** The nature of the specific interactions are given in a red–blue–green colour scheme, which indicate strong attractive, strong repulsive and weak interactions, respectively. The blue isosurface near H1 and N1 confirms the strong attraction associated with the O1—H⋅⋅⋅N1 hydrogen bond but red isosurfaces inside the benzene and cyclohexyl rings indicate strong repulsion forces. Weak interactions are also observed around atom H2.

**Reference**

Humphrey, W., Dalke, A. & Schulten, K. (1996). *J. Mol. Graph.* **14**, 33–38.

**Figure S2** Powder X-ray diffraction (PXRD) patterns comparing (a) the calculated pattern from single crystal data, and (b) the experimental PXRD data of (I).

**Spectroscopic characterization and powder diffraction**

Fourier transform infrared spectrum of the title compound (I) was measured at a spectral resolution of 4 cm^-1^ by a Bruker Equinox55/S spectrophotometer equipped with deuterated triglycine (DTGS) detector under constant purge with dry air. The sample of the title compound (I) was ground thoroughly with dry KBr (spectroscopic grade from Fisher) and then uniaxially-pressed into a pellet before measurement. Raman spectrum of the title compound (I) was determined by a high-resolution Raman microscope spectrometer, HORIBA Scientific, LabRAM HR evolution). Three areas of the title compound (I) are used for the analysis to confirm the homogeneity of the sample. Nuclear magnetic resonance (NMR) spectrometer (Bruker AVANCE III 500 MHz for ^1^H and 126 MHz for ^13^C) was used to perform ^1^H-NMR and ^13^C-NMR studies. The titled compound (I) was dissolved in CDCl_3_ prior to the NMR investigation. Elemental analysis (EA) was performed by a Perkin Elmer 2400 Series II CHNS/O analyzer with a combustion temperature of 975 °C and a reduction temperature of 500 °C.

Powder X-ray diffraction (PXRD) data of the title compound (I) was collected at Beamline 1.1W (Multiple X-ray Techniques), Synchrotron Light Research Institute, Thailand, using the monochromatic synchrotron X-ray radiation of the energy 12 keV (wavelength of 1.0332 Å). The crystals of the title compound (I) were firstly ground to obtain fine powder. Then the fine powder sample was packed in Kapton capillary with a diameter of 0.5 mm and aligned using a goniometer head. During the XRD measurement with a setup in Debye-Scherrer geometry, the capillary was constantly rotated. The diffraction pattern was recorded by a strip detector (Mythen6K 450, Dectris®) in the 2θ range of 5-40° (with respect to the X-ray energy of 12 keV). The observed XRD pattern was compared to the calculated XRD pattern generated by the single crystal X-ray diffraction data by employing the Mercury software.
